# Perceptions of Community Health Workers (CHW) on barriers and enablers to care for people with psychosis in rural Mozambique: findings of a focus group discussion study using the Capability, Opportunity, Motivation and Behaviour framework (COM-B framework)

**DOI:** 10.1186/s12960-022-00741-0

**Published:** 2022-05-19

**Authors:** Dirceu Mabunda, Déborah Oliveira, Mohsin Sidat, Francine Cournos, Milton Wainberg, Jair de Jesus Mari

**Affiliations:** 1 Department of Psychiatry and Psychological Medicine, Universidade Federal de São Paulo, São Paulo, Brazil; 2 Division of Social Protection and Health, InterAmerican Development Bank, Washington, DC United States of America; 3 Department of Community Health, Faculty of Medicine, Eduardo Mondlane University, Avenue Salvador Allende nr. 702, P.O Box: 1106, Maputo, Mozambique; 4 Department of Psychiatry, Columbia University College of Physician and Surgeons, New York, NY United States of America

**Keywords:** Community health workers, Psychosis, Enablers, Barriers, Capacity, Opportunity, Motivation, Behaviour

## Abstract

**Background:**

Psychotic disorders contribute significantly to the global disease burden by causing disability, impaired quality of life, and higher mortality in affected people compared with the general population. In rural settings, where there is limited or no access to healthcare, individuals living with psychotic disorders often seek support from Community Health Workers (CHWs). However, little is known about what CHWs know about psychosis and how they manage such cases. This study aimed to explore the CHWs perception of psychosis and their experiences and beliefs about the factors that might enable or hinder care-taking for patients with psychosis in rural settings in Mozambique.

**Methods:**

A qualitative study was conducted in rural districts of Maputo Province, a southern region of Mozambique, using six focus group discussions with participation of 79 CHWs. Thematic analysis was used informed by the Capabilities, Opportunities, Motivation and Behaviour framework (COM-B).

**Results:**

Nine primary themes were identified. Overall, CHWs perceived psychosis as treatable medical conditions and held a positive attitude about being part of the care-taking process of patients with psychosis in rural settings. Partnerships with key-stakeholders such as traditional healers, health care workers, and families, were perceived by CHWs as enablers to improve access to care in rural areas. However, stigma, myths, and lack of competencies to treat people with psychosis were perceived by CHWs as barriers for appropriate care.

**Conclusion:**

CHWs, with adequate support, could play an important role in the care of patients with psychosis in rural settings, including identifying patients requiring care and referring them to appropriate healthcare professionals, and following up medicated patients with psychosis. Training of CHWs should consider inclusion of basic mental health care competencies.

**Supplementary Information:**

The online version contains supplementary material available at 10.1186/s12960-022-00741-0.

## Introduction

Psychotic disorders contribute significantly to the global disease burden by causing disability, impaired quality of life, and higher mortality in affected people compared with the general population [1, 2]. Mental disorders increased by 48.1% in the world from 645.8 million cases in 1990 to 970.1 million cases in 2019. Schizophrenia affects a lower proportion of the world's population compared to depression and anxiety, but the estimate of the weight of disability due to the acute symptoms of psychosis was greater among all mental disorders[3]. Schizophrenia is the main cause of psychosis and has been identified as a priority disorder by the Global Mental Health Initiative [4]. Though the exact causes of schizophrenia are unknown, it is believed that this illness is generated through the interaction between genetically determined characteristics that influence brain structure and function, with environmental stressors from prenatal (delivery complications, maternal infections) to post-natal events (post-natal brain trauma) [5]. Nevertheless, the median treatment gap for schizophrenia in low and middle-income countries is approximately 70% [6] and a scarcity of human resources for the treatment of psychosis has been identified as a contributor to this existing gap globally [7].

Mozambique is a low-income country where about two-thirds of its total population (32 million people in 2020) live in rural areas. In Mozambique, the prevalence of psychosis is regarded as being higher in rural compared to urban areas [8]. Schizophrenia is the leading cause of inpatient admission to psychiatric hospitals and the second most common reason for psychiatric outpatient consultation [9]. Family support, community-based rehabilitation, self-help and support groups are some of the key priority interventions to address the burden of mental, neurological and substance use disorders in people living with psychosis [10]. Interventions delivered by Community Health Workers (CHW) in India suggest that they can effectively reduce disability and increase adherence to treatment in people living with schizophrenia [11]. The effectiveness and feasibility of community-based interventions for people living with psychosis have been demonstrated in Chile [12], India [13], Nigeria and Ghana [14].

The CHW programme (*Programa dos Agentes Polivalentes Elementares-APEs*) in Mozambique was created in 1978, informed by the primary health care approach, with the aim of expanding access to health care in remote and rural regions [15]. Training to APEs is provided by local health care providers with supervision of local health officials during a period of 18 weeks in total. Training includes health promotion, disease prevention, first aid, and management of common diseases. APEs receive a monthly stipend of US$20 (equivalent to 35% of national minimum wage) provided by the Ministry of Health and are expected to have another complementary means of income to sustain their economic needs. In addition, they receive a kit which includes the following: a bicycle, a flashlight, a vest, a medicine bag, an identification badge, a hat, a calculator, a thermometer, and a stopwatch [16]. After a challenging period followed by a civil war in the country (1977–1992), the programme was relaunched in 2010 with a defined training package, supervision strategies and logistical support [17]. However, the mental health and neurological component is still inadequately addressed, with only a brief reference to epilepsy [18]. Many people with psychosis will start treatment elsewhere and then return to their communities requiring continued attention and follow-up. In rural areas, this attention is scarce or non-existent. We focused on CHWs because they are important health care providers in rural areas of Mozambique [19]. Having CHWs as providers of mental health interventions for people living with psychosis in rural areas could potentially contribute to narrowing the gap in mental health care in Mozambique. However, studies about the knowledge and perceptions of CHWs on the barriers and enablers of mental health care for people living with psychosis in Mozambique are scarce.

## Aim

The study aimed to explore the CHWs perception of psychosis and their experiences and beliefs about the factors that might enable or hinder care-taking for patients with psychosis in rural settings in Mozambique.

### Theoretical framework

The model of behaviour change “Capability, Opportunity, Motivation and Behaviour” (COM-B) [20] was used to inform our focus group guide and data analysis. Our goal was to better understand the interactions between capability, opportunity and motivation on behaviour that could enable access to health care. We used the COM-B framework because this theoretical framework has been applied widely to identify barriers and enablers to behaviour change and to inform the design of interventions within mental health settings [21–23]. COM-B can be used to facilitate the design of interventions [20] aimed at improving access to healthcare by people living with psychosis in rural settings of Mozambique. In this framework, capability includes the necessary knowledge and skills that enable a person’s psychological and physical capacity to engage in an activity. Motivation includes the brain processes that enable direct behaviour, including habitual processes, emotional responses and analytical decision-making. Opportunity encompasses all the external factors which make the behaviour possible.

## Methods

### Study design

A qualitative study was carried out and data collected through focus group discussions with CHWs. The focus groups were regarded as appropriate as they allowed for the probing of specific issues in group interaction and clarification of individual and shared perspectives of health issues [24]. This sharing of experiences enhances additional thoughts and insights between group discussion participants (CHWs), providing opportunities to make connections and learn from shared experiences during the group dynamics.

### Study setting

Three rural districts of Maputo province in southern region of the country were included: Manhiça, Moamba and Boane.

*Manhiça* district is located in the north area of Maputo Province, 80 km from Maputo City. It has about 214 751 inhabitants. It has 15 primary care units and 41 CHWs. *Moamba* district is located to the north area of Maputo Province, 52 km from Maputo City. It has about 56 559 inhabitants. It has 11 primary care units and 32 CHWs. *Boane* district is in the south-eastern region of Maputo Province, 30 km from Maputo City. It has about 134 006 inhabitants. It has 10 primary health care units and 12 CHWs. Each CHW provides care for between 500 and 2000 community members in an 8 to 25-km radius from the local health facility [25].

### Sample and recruitment

Using a purposive sampling strategy [26], we identified and subsequently invited all CHWs from the three districts to participate voluntarily. The participants were included in each FGD in order of appearance. Those that agreed were additionally informed about the time and place where the focus group discussions would occur. Recruitment was led by DM and took place during monthly district meetings at district health clinics between September and December 2019. Travel expenses were reimbursed, but no other incentives were offered. The objectives of the study were explained to the CHWs who agreed to participate, and informed consent was obtained. Discussions were facilitated in the Portuguese language by DM, a male researcher trained in qualitative methods with no previous contact with the participants. Each session was audio-recorded and transcribed verbatim by DM. In addition to these recordings, written notes were also taken, to allow for future review during data analysis and other research processes and to ensure that participants views were clearly documented.

### Procedure

The study was approved by the National Review Board (92/CNBS/2017) and all participants signed the informed consent prior to the focus group discussion. The focus group guide was designed by the research team and informed by the COM-B framework. Before the study commenced, the focus group guide was piloted in other districts which were not part of the study, so that it could be refined and revised. Because CHWs are not trained in mental health themes, we adapted the mhGAP case vignette of psychosis [27] to provide a unified description of the common experiences of psychosis. Participants were asked to identify if they have had previous experiences with similar cases and identify the causes (Additional file 1).

### Data analysis

Transcripts were analysed manually as we had a diverse team involved in the coding process and not all of them were familiar with the use of qualitative softwares using a word template based on guidelines for the thematic analysis [28]. The following steps were taken to analyse the data: (1) familiarization: the first author (DM) revisited the focus group discussion audio and verbatim transcript several times to familiarize himself with the data; (2) code generation: the coding was derived from the data using the structural coding approach [29]; (3) theme search: after initial coding, a set of overarching themes were developed; (4) theme revision: revision of the coding process was performed, during which themes were refined and new themes could be added; (5) theme definition and naming: consultation with a second researcher (DO) was undertaken to refine the way each theme was developed; and (6) thematic final map: this map was used to inform the reporting of the analysis. Data saturation was assessed following Monike Hennik’s guidelines on saturation [30] which states that a study where focus group are not stratified by any characteristic will require a small sample size to reach saturation (3–6 groups). Reporting followed the Consolidated Criteria for Reporting Qualitative Research [24].

## Results

Seventy-nine representative CHWs took part in six focus groups (group size *n* = 5–13), and each focus group lasted between 65 and 100 min. The socio-demographic characteristics of the sample are described in Table 1.

**Table 1:** Socio-demographic characteristics of participants

Variable	Focus group (*n* = 79) Mean (SD)
Age (years)	38.81 (11.92)
Time as CHW (years)	7.71 (6.91)
Gender *n* (%)	
Male	28 (35.4)
Female	51 (64.6)
District	
Boane	13 (16.5)
Manhiça	34 (43)
Moamba	32 (40.5)
Education	
Primary	36 (45.6)
Basic	26 (32.9)
Secondary (12 years)	7 (8.9)
No information	10 (12.7)
Marital status *n* (%)	
Single	53 (67)
Married	24 (30.5)
Divorced	2 (2.5)

*CHW* Community Health Workers, *SD* standard deviation, *n* number, % percentage

Nine primary themes were identified: knowledge about psychosis and mental health; the manifestations of the disease; attitudes and practices towards patients with psychotic disorders; key-stakeholders in the care of patients with psychotic disorders; perceived needs for training; perceived readiness to provide care for patients with psychosis; community perceptions; and beliefs and attitudes related to psychosis. Table 2 shows details of COM-B model related themes.

**Table 2:** COM-B model (Michie et al., 2011) and related themes

Enablers and barriers to care for people with psychosis	COM‐B components	Definitions	Themes
	**Capability:** “the individual's capacity to engage in the activity concerned”	Psychological capability: ‘the capacity to engage in the necessary thought processes e.g.: comprehension, reasoning, knowledge.’	1-Knowledge about the cause of psychosis (enabler) 2-The nature of the disease (barrier)
		Physical capability: physical skills to enact the behaviour. e.g.: skills, ability, proficiency acquired trough practice	3-Training need (barrier)
	**Opportunity:** ‘the factors that lie outside of the individual that make behaviour possible or prompt it’	Social opportunity: ‘afforded by the cultural milieu’ which ‘dictates the way we think about things. Eg: social influences, norms, conformity, social comparisons	4-Attitude and practice of CHWs (enabler) 5-Partnership (enabler) 6-Engage families in care pathway (enabler/barrier) 7-Stigma (barrier)
	**Motivation:** ‘all those brain processes that energize and direct behavior’	Reflective motivation: ‘analytical decision-making’, ‘reflective processes (involving evaluation and plans)’ e.g.: beliefs about capability and consequences, roles, identity, intentions, goals	8-Lack of confidence (barrier)
		Automatic motivation: ‘involving emotions and impulses that arise from associative learning and/or innate dispositions. E.g.: emotions, reinforcement such as rewards, incentives, punishments	9-Readiness of CHW to provide mental health care (enabler)

### Capability

Capability can be explained as the CHW’s capacity to engage in the management of mental health issues for people with psychosis in rural settings. Two barriers and one enabler emerged in relation to this construct. Three themes were identified within the category of capability: knowledge about the cause of psychosis, understanding of the nature of the disease, and lack of mental health skills.

### Knowledge about cause of psychosis (enabler)

To determine levels of knowledge about psychosis, participants were asked to define the causes of psychosis. Generally, CHWs demonstrated limited knowledge and understanding of psychosis. Most CHWs indicated that alcohol use and substance abuse can be important causes of psychosis or worsening of symptoms. The theme related to knowledge about the causes of psychosis encompassed 5 sub-themes: alcohol and substance abuse; malaria infection in childhood; family inheritance; young age; and family problems, such as trauma (sexual trauma, stress, anger), bad spirits, punishment, and isolation (discrimination, orphaned in childhood).

The main substance implicated in the development of symptoms by CHWs was perceived to be *Cannabis sativa* “*suruma*”:

“To my mind, such illness strikes young people [the most], since most young people use suruma a lot, so many are there due to their using of suruma.” FGD1, 01

One CHWs thought that psychosis is caused by heritability from parents to children, besides the use of drugs.

“To my mind, these problems are rooted in the families, perhaps. Suppose, perhaps, there are families who have had cases of that illness, and maybe they think that such persons would pass it on to others.” FGD 2 07

Few CHWs believed that psychosis is caused by witchcraft and possession by bad spirits:

“In most-people’s minds, this is witchcraft, in other words, someone has been bewitched...the community thinks that this illness is rooted in tradition.” FGD 2 03

One CHW observed that psychosis could be the result of malaria infection during childhood, but did not discard the possibility of psychosis being the result of “evil spirits”:

“I can’t tell, and scientifically speaking, it is difficult to tell. Still, it seems that a person, in particular kids, for instance, who had cerebral malaria earlier, right, can develop this illness, scientifically speaking… I cannot tell whether or not myths become a reality, but what people have said is that these illnesses have to do with evil spirits.” FGD 5, 03.

Discrimination and lack of empathy was stated as a cause of psychosis by one CHW:

“Consider the example of someone who is discriminated where they live, they may eventually suffer from this illness. If he/she feels lonely without being hugged by their relatives, he/she may develop this illness.” FGD 2 06

### Training needs (barrier)

CHWs felt that they lacked the skills needed to care for people with psychosis and suggested practical and theoretical training to overcome this gap:

“We are not yet well prepared on the topic, right? Given that we are not knowledgeable about the topic, we shall be prepared, well prepared after undergoing a small training.” FGD 06 8

### The nature of the disease (barrier)

Participants considered that the ability to provide care for people with psychosis could be influenced by the nature of the disease as patients are often unaware of their symptoms and this can affect how CHWs and families provide care (or how prepared they feel to provide care), e.g. how they can communicate with the person and how the person will respond. One CHW recognized that it is difficult to deal with people with psychosis because of their symptoms and behaviour:

“Someone with mental disorders can, for instance, serve food on a plate, then dump it onto the floor, collect the food from the floor and eat it. All that is normal is seen by them as something abnormal, as I explained earlier.” FGD 05 6

### Opportunity

Two enablers emerged as influencing CHWs’ opportunities within this construct: attitudes and practices of CHWs towards psychosis and partnerships. Engaging families in the care pathway was identified as a barrier as some families do not seem to understand that this illness could be treated in the hospital. An enabler is that CHWs can advise and support families in the existing care pathway. Stigma is another barrier in the social opportunity domain as the stigmatizing social norms, influences and beliefs may hinder access to care. Negative perceptions and attitudes about psychosis may increase stigma and hinder the access to care.

### Attitude and practices of CHW (enabler)

Seven CHWs in three FGD felt that are empowered and motivated to care for people living with psychosis:

“But we as CHWs have been entrusted by the community with working for them. We already have several types of packages with which we are working. We’re going to give the case a try until we succeed, because that person belongs to the community, that is a person of ours.” FGD 1 04

### Partnerships (enabler)

CHWs reported that partnering with families, traditional healers and health care workers was a way to improve access and care for people living with psychosis in their communities as CHWs perceived that the family members of the clients believes that mental illness can only be treated by traditional healers:

“Because when this sort of illness appears, families think that this illness can’t be cured at the hospital, but that the illness can only be cured by traditional healer(s). We are not refusing this. We are saying yes, you can go and see traditional healers, don’t stop seeing them, but first go to the hospital.” FGD 01 6

“Our role is to raise the awareness of that ill-person’s family, give that family advice by stating that person who is ill should be taken to the hospital, where the person will get treated. If by any chance he comes with medicines, we will endeavor to enable that the medicine is given to that ill person.” FGD 01 11

### Engage families in care pathway (enabler/barrier)

Almost all CHW recognize that they have a challenge in convincing families to engage in the care of their children with psychosis. They also recognize that families need support from the community and all stakeholders to fulfil the needs of people living with psychosis:

“It’s not easy for the community to understand that this illness can be treated at the hospital, but whenever a visit is paid by us we should give advice, give support so that they take him/her to the hospital, to see whether they can eventually understand it. He/she will end up accepting the pieces of advice and whenever he/she is taken to hospital he/she will see that things will change.” FGD 05 2

Education and providing support for people with psychosis and their caregivers were seen by CHWs as enablers for recovery:

“As for my community, I, as a CHW, have to help those people who do not understand that the illness can be treated at the hospital, advise the ladies or the families to take the person with that disability to hospital, in order to get better…” FGD 02 8

CHWs are able to support the communities to better understand the causes of psychosis and increase awareness about mental health issues:

“We have to make more auxiliary visits to help our communities. The communities don’t know where else that illness comes from… As CHWs we have to help the community by raising their awareness so that community members can also know that a person can get ill and not be in a spell, but that at the hospital he/she can be cured.” FGD2 02

CHWs stated that families who care for people with psychosis are reluctant to go to clinic because of financial difficulties and that they are not comfortable with continuing through the complex referral and treatment pathway:

“Yes, because the difficulties are the fact that communities already share the conviction that this kind of illness is not treated at a hospital. That is the most common difficulty, all the more so because they tell us that if they go there, they are going to be sent to Magude, and from Magude, they will be sent to Maputo. Thus, they don’t have money to go there.” FGD 02 8.

### Stigma issues (barrier)

Stigma issues were recognized by CHWs as barriers for access, diagnosis, recovery and continuity of care by people living with psychosis. CHWs felt that some families of people living with psychosis treat them with dehumanization and blame:

“I felt very sorry when two months ago a boy who is still old enough to go to school or do what is for his own good, received an unsatisfactory response from his father towards his illness, as the father tied him up and beat him up a lot and put him on his minibus.” FGD 1 04

Families in the rural communities may express their stigma about psychosis by sanctioning violent acts against people manifesting psychotic symptoms. One CHW stated that people do not believe that mental disorders exist. They think that it is a personal choice to have a mental illness:

“As far as recovery is concerned, the difficulty lies with the family itself, because when the family sees the child in the state he/she is messing up everything, they think he/she only does that because he/she wants to [do so], but no, that is an illness.” FGD 1 04

The CHWs also recognized that misinterpretation about the origin/causes of the psychosis may negatively impact recovery and create barriers to accessing mental health services and care and that stigma is often generated due to lack of knowledge or negative beliefs about the condition: “The communities don’t know where else that illness comes from. People say that thing that somebody got bewitched. The thing is that there are people who say that cure can’t be found there, you can only go to a traditional healer and be given I don’t know what.” FGD2 02.

CHWs felt discriminated against by some community members because they are members of the same community and mental health themes are not part of their current intervention package:

“Another thing involves the community: It is the fact that we were born there, grew up there, are known by people, people know what we do in our homes, people are aware that we are poor. So, when we approach the community, they don’t take us seriously—they think that we are lying; They despise us.” FGD 02 10

“For example: now if I come up, the family will ask what it is that I bring anyway. They will say that I’m lying, and then the community will feel doubtful wondering how come that person, as a CHW who should treat malaria, today he is treating mental illnesses. They will say that they know that mental illnesses are treated at the hospital.” FGD 01 7.

### Motivation

One barrier and one enabler were categorized as influencing participants’ motivation to manage people with psychosis.

Some (7 CHWs in 3FGD) CHWs felt motivated and prepared for their role, with a positive attitude towards providing mental health care for people with psychosis.

### Lack of confidence (barrier)

CHWs perceived a lack of confidence and skills/ability to care for people with psychosis and their families as barriers. This reduces the self-efficacy of CHWs to care for people with psychosis. Building that capacity and professional competence were seen as important factors for CHW motivation:

“As we do not know enough about the illness, if we approach a family to offer explanations, family members in the first place would want to know what is happening. These are questions which, at that moment, we would not be able to answer. And our credibility as CHWs there is virtually nil.” FGD 06 12”.

Because CHWs so far have not addressed mental illness in the community, CHWs felt that households where a family member is a person living with psychosis would not trust them as a source of knowledge:

“Again, we CHWs are not known there in the community as persons dealing with these stuff, except for dealing with malaria, cholera, diarrhoea. Meanwhile, nowadays, if we approach them with this topic, they will ask us where we got this information from, and that we are lying.” FGD 01 4

### Readiness of CHW to provide mental health care (enabler)

CHWs perceived that they would be able to raise awareness within the community and among families of people with psychosis in a way that increases knowledge about psychosis. Additionally, they felt that they were able to care for patients with psychosis and help their communities:

“But we as CHWs have been entrusted by the community with working for them. We guarantee that we will do what we have been doing with those other packages, because so far we have already been achieving the goals. We’re not going to set someone aside in that it’s difficult. We’re going to give the case a try until we succeed, because that person belongs to the community, that is a person of ours.” FGD 1 04

## Discussion

This qualitative study aimed to explore the CHWs’ perceptions of psychosis and their experiences and beliefs about the factors that might enable or hinder care-taking for patients with psychosis in rural settings in Mozambique. It sheds light on how CHWs understand psychotic illnesses, their desires for further education and skills to improve their understanding and how they approach psychotic illnesses, and how a lack of knowledge and misunderstandings about the causes of psychosis in these rural communities hampers appropriate approaches to deal with stigma and prevent abuse of people with psychosis.

Our findings suggest that CHWs have a good attitude and perceive themselves as being ready to provide mental health care for people with psychosis and that they are available to be trained to perform this additional task. Furthermore, CHWs also consider that having a good collaboration between CHWs, families, traditional healers and health care professionals is an important enabler to engage/maintain people with psychosis with appropriate care while residing in remote rural settings. The transfer of tasks from highly qualified health care professionals to CHWs—task shifting, has been a leading strategy to address the shortage of human resources in health and expand the access to health services [31]. For task-shifting to occur, the lower cadres (such as CHWs) need to accept and be ready for it.

The study indicates that CHWs need to have appropriate knowledge about psychosis as this is likely to contribute towards more positive attitudes and practices towards people living with psychosis. Many CHWs were aware of factors that may contribute to the onset of psychotic illnesses, such as the abuse of alcohol and illicit substances, especially marijuana, the role of cerebral malaria in contributing to mental illness, and the presence of other contributing factors, such as trauma. CHWs were able to discuss community beliefs about witchcraft and spirit possession as factors that needed to be addressed appropriately to promote better acceptance of psychiatric treatment in rural communities. These findings are in line with other studies [32].

A systematic review suggests that family and community support for people with psychosis results in good outcomes and recovery [33]. In this study, family support was stated as an important enabler to recovery of people with psychosis. This was also suggested in previous studies [34, 35]. CHWs in this study faced issues of lack of knowledge and skills to care for people with psychosis and suggested training in mental health themes. Our study is in line with other studies that were carried out in Kenya about the helpfulness of interventions that improve knowledge among CHWs through training on the diagnosis of psychosis [36]. Our study is the first in Mozambique to explore behaviours and attitudes towards mental health service provision in the perspectives of CHWs and highlighted that there is an opportunity to incorporate mental health care for CHWs in Mozambique which is sustainable and may have a good impact in reducing the treatment gap through increasing access to mental health care in remote areas of Mozambique. A community-based comparative study in Brazil integrated CHWs more closely with mental health professionals as supervisors in *Centros de Atenção Psicossocial (CAPS),* and in Chile the CHWs worked independently in the community and neighbourhoods [37]. These experiences might be useful to our context as these interventions could be adapted and used for similar purposes.

A randomized controlled trial in Nigeria and Ghana suggested that collaborative care between traditional healers and primary healthcare workers was more effective and cost-effective for care for people with psychosis in low resource settings [14].

The goal of our study was to explore the views of CHWs concerning the barriers and enablers of access to mental health services and recovery services for people with psychosis in their communities. Our findings shed important light on the negative impact of the stigma on access to mental health care and recovery in rural areas of Mozambique. They suggest the need for improved collaboration/partnerships between families, traditional healers, CHWs and health care workers.

A study from Kenya suggested that discrimination related to psychosis was an important barrier to service access that contributed to low levels of knowledge towards psychosis among CHWs [36].

Additionally, CHWs found it challenging to provide care for people with psychosis, but they also felt many barriers could be lowered by training CHWs on mental health themes and on techniques for engaging/convincing families to be involved in the care pathway for people with psychosis. One previous study found that training for CHWs, along with providing family support and community mobilization, had a positive impact on the quality of life of people with schizophrenia [38]. Some CHWs felt they would be discriminated against by their communities in case they provided mental health care, highlighting the impact of stigma on those who are caring for people with psychosis. This exacerbates stigma as it associates providing care for people with psychosis with low-prestige activity.

### Limitations

The sample was composed by CHWs of the southern region of Mozambique and there are considerable ethno-cultural differences between different parts of the country which limit the applicability of findings to the whole of the country. These CHWs’ suggestions and opinions may not reflect those of CHWs in other contexts elsewhere in Africa or globally. However, this study is the first to explore the barriers and enablers of mental health care for people with psychosis living in rural settings in Mozambique from the perspective of CHWs. This study can serve as a background for further research focusing on the feasibility of implementing evidence and community-based interventions for people with psychosis in rural areas of Mozambique. The participation of CHWs in the care of patients with psychosis is of paramount importance as they are the ones who deliver basic health care and are the first point of contact with the health system in the rural areas of Mozambique.

## Conclusion

In our study, CHWs were willing and felt ready to provide screening, referral and follow-up for people with psychosis in rural settings. In conclusion, this study presents exploratory evidence of the roles that CHWs within the rural remote setting could play in participating in care of patients with psychosis. CHWs, with adequate support, could assist with identifying patients requiring care, referring patients to appropriate healthcare professionals, following up medicated patients with psychosis, and working with affected families to improve understanding of psychosis and its treatment. Training of CHWs should consider inclusion of basic mental health care competencies.

## Supplementary Information

**Additional file 1: Table S1.** Topic guide. **Table S2.** Consolidated criteria for reporting qualitative studies (COREQ) 32-item checklist.

## Data Availability

The data are available under reasonable request addressed to Dirceu Mabunda (dihepama@gmail.com).

## Abbreviations

CHW: Community Health Workers
COM-B: Capability, Opportunity, Motivation and Behaviour
*APE*: Agente Polivalente Elementar
*CNBS*: Comitê Nacional de Bioética em Saúde
FGD: Focus group discussions
