# Supplementary material for: Perceptions of Community Health Workers (CHW) on barriers and enablers to care for people with psychosis in rural Mozambique: findings of a focus group discussion study using the Capability, Opportunity, Motivation and Behaviour framework (COM-B framework)
Source: Hum Resour Health. 2022 May 19;20:44. doi: 10.1186/s12960-022-00741-0 (PMC9118750; doi:10.1186/s12960-022-00741-0)
Supplement: Supplementary file 1 — Additional file 1: Table S1. Topic guide. Table S2. Consolidated criteria for reporting qualitative studies (COREQ) 32-item checklist. [file 12960_2022_741_MOESM1_ESM.docx]

**Supplementary Materials**

Recruitment took place within the district health directorate during the monthly meeting of the CHWs. Focus groups with the CHWs were planned to ensure that experiences of rural and community health workers were included. CHWs received oral and writen informed consent with details of the research methodology and aims of the research.

The topic guide was developed by MS (Associate Professor, with qualitative methods experience), JM (Associate Professor with experience in qualitative research) and DM (PhD student with training in qualitative methods). Focus groups were led by DM and two colleagues from the Department of Community Health at Faculty of Medicine- University Eduardo Mondlane. All participants were asked to keep their responses confidential and not discuss them with one another outside of the focus groups. No one who had previously agreed to participate withdrew from the groups. Focus groups in the three districts were held at the District Health Directorate Office.

One researcher (DM) undertook the familiarisation and initial coding stages. Each transcribed focus group was read fully at least once to be as familiar as possible with the data before formally coding. Having re-read the transcripts, an initial list of codes was produced. Codes were revised during the analysis process. The codes and process used to reach them, and development and review of interpretive themes, was then discussed and verified with other researchers in the team.

**Table S1. Topic guide**

| **Steps** | **Questions for the CHW** |
| --- | --- |
| **Opening** | *Please introduce yourself with your name and professional background.* |
| **Introduction** | Presentation of the case vignette about a patient with psychosis.  **Case vignette:** “Jota is 21 years old and has been brought to you by his mother. His mother says that recently Jota "is not the same." He is no longer studying and prefers to stay home doing nothing. You notice that Jota is wearing summer clothes although it is cold and raining. He looks like he has not washed for weeks. When you talk to him, Jota avoids eye contact. He gazes at the ceiling as if looking at someone. He mumbles and gestures as if he is talking to someone. He does not want to see his friends; he seems disconnected from his family and has no energy. He is refusing to eat food in the home as he believes his mother is trying to poison him.” |
| **Capacity** | 1. Have you seen a patient with these symptoms?  2. Have you ever treated a patient with these symptoms?  3. What is your experience regarding the management of patients with psychotic disorders?  4. What, in your opinion, are the causes of psychotic disorders? |
| Opportunity | 1. What helps and what makes the recovery process of patients with psychosis difficult?  3. What are the challenges you face in caring for a patient with a psychotic disorder?  4. What is the role of family and friends in the recovery process of the patient with psychotic disorder?  5. What should be the role of existing services in the community in the recovery process for patients with psychotic disorders? |
| **Motivation** | 1. What do you think you can do to help in the care of patients with psychotic disorders?  2. In your opinion, where do you think patients with psychotic disorders should be treated? And who should offer this care?  3. Is information about mental health / psychotic disorders available in convenient places in your health facility / community? |

**Table S2. Consolidated criteria for reporting qualitative studies (COREQ) 32-item checklist**

| Topic and Item No. | Guide Questions/ Description | Reported on Page No. |
| --- | --- | --- |
| Domain 1: research team and reflexivity | | |
| Personal characteristics | | |
| Interviewer/facilitator | Which author/s conducted the interview or focus group? |  |
| Credentials | What were the researcher’s credentials? E.g. PhD, MD | Supplementary materials and title page |
| Occupation | What was their occupation at the time of the study? | 4 |
| Gender | Was the researcher male or female? | 4 |
| Experience and training | What experience or training did the researcher have? | 4 |
| Relationship with participants |  |  |
| Relationship established | Was a relationship established prior to study commencement? | 4 |
| Participant knowledge of the interviewer | What did the participants know about the researcher? | 4 |
| Interviewer characteristics | What characteristics were reported about the interviewer/facilitator? | 4 |
| Domain 2: study design | | |
| Theoretical framework | | |
| Methodological orientation and Theory | What methodological orientation was stated to underpin the study? | 3 |
| Participant selection | | |
| Sampling | How were participants selected? | 4 |
| Method of approach | How were participants approached? | 4 |
| Sample size | How many participants were in the study? | 4 |
| Non-participation | How many people refused to participate or dropped out? Reasons? | 4 |
| Setting | | |
| Setting of data collection | Where was the data collected? | 4 |
| Presence of non-participants | Was anyone else present besides the participants and researchers? | 4 |
| Description of sample | What are the important characteristics of the sample? | 4, 5 |
| Data collection | | |
| Interview guide | Were questions, prompts, guides provided by the authors? Was it pilot tested? | 4 |
| Repeat interviews | Were repeat interviews performed? If yes, how many? | N/A |
| Audio/visual recording | Did the research use audio or visual recording to collect the data? | 4 |
| Field notes | Were field notes made during and/or after the interview or focus group? | 4 |
| Duration | What was the duration of the interviews or focus group? | 4 |
| Data saturation | Was data saturation discussed? | 5 |
| Transcripts returned | Were transcripts returned to participants for comment and/or correction? | Nil |
| Domain 3: analysis and findings | | |
| Data analysis | | |
| Number of data coders | How many data coders coded the data? | 5 |
| Description of the coding tree | Did authors provide a description of the coding tree? | Supplementary |
| Derivation of themes | Were themes identified in advance or derived from the data? | Themes derived form data |
| Software | What software, if applicable, was used to manage the data? | N/A |
| Participant checking | Did participants provide feedback on the findings? | Nil |
| Reporting | | |
| Quotations presented | Were participant quotations presented to illustrate the themes / findings? Was each quotation identified? | Table 2 |
| Data and findings consistent | Was there consistency between the data presented and the findings? | Yes |
| Clarity of major themes | Were major themes clearly presented in the findings? | Table 2 |
| Clarity of minor themes | Is there a description of diverse cases or discussion of minor themes? | Table 2 |
